# (5,5′-Dimethyl-2,2′-bipyridine)­iodido­trimethyl­platinum(IV)

**DOI:** 10.1107/S1600536811014085

**Published:** 2011-04-22

**Authors:** Fredrik Lundvall, David Stephen Wragg, Mats Tilset

**Affiliations:** a Centre for Materials Science and Nanotechnology, Department of Chemistry, University of Oslo, PO Box 1126, 0315 Oslo, Norway; b Centre for Materials Science and Nanotechnology & inGAP National Centre of Research-based Innovation, Department of Chemistry, University of Oslo, PO Box 1126, 0315 Oslo, Norway; c Centre of Theoretical and Computational Chemistry (CTCC), Department of Chemistry, University of Oslo, PO Box 1033, 0371 Oslo, Norway

## Abstract

In the title compound, [Pt(CH_3_)_3_I(C_12_H_12_N_2_)], the Pt^IV^ atom is six-coordinated in a slightly distorted octa­hedral configuration with one CH_3_ group and the I atom forming a near perpendicular axis relative to the square plane formed by the bipyridine ligand and the two remaining CH_3_ groups. The CH_3_ group *trans* to the I atom has a slightly elongated bond to Pt compared to the other CH_3_ groups, indicating a difference in *trans* influence between iodine and the bipyridine ligand.

## Related literature

For synthetic background to related complexes containing Pt(CH_3_)_3_, see: Clegg *et al.* (1972 ▶); Vetter *et al.* (2006 ▶). For structural information on complexes exhibiting a similar geometrical configuration around the Pt^IV^ atom, see: Hambley (1986 ▶); Hojjat Kashani *et al.* (2008 ▶); Vetter, Bruhn & Steinborn (2010 ▶); Vetter, Wagner & Steinborn (2010 ▶). For examples of bimetallic metal-organic frameworks (MOFs), see: Bloch *et al.* (2010 ▶); Szeto *et al.* (2006 ▶, 2008 ▶).
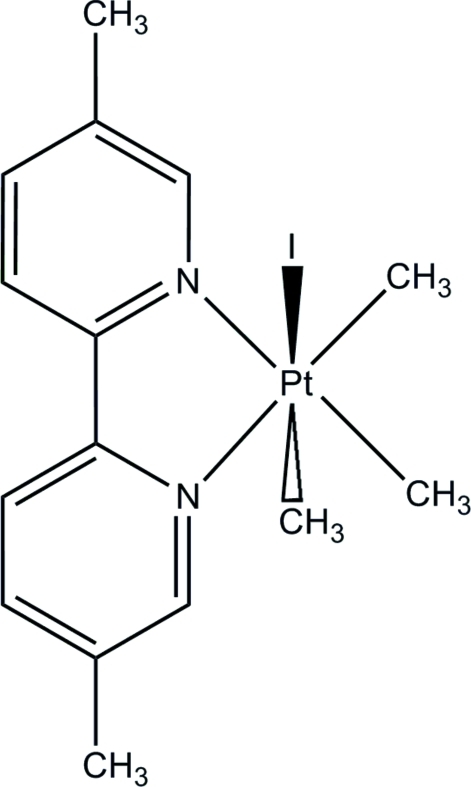


## Experimental

### 

#### Crystal data

[Pt(CH_3_)_3_I(C_12_H_12_N_2_)]
*M* *_r_* = 551.32
Monoclinic, 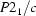

*a* = 15.354 (3) Å
*b* = 12.394 (2) Å
*c* = 9.0627 (18) Å
β = 106.222 (2)°
*V* = 1655.8 (6) Å^3^
*Z* = 4
Mo *K*α radiation
μ = 10.33 mm^−1^
*T* = 293 K
0.4 × 0.4 × 0.1 mm

#### Data collection

Bruker APEXII CCD area-detector diffractometer
Absorption correction: numerical (*SADABS*; Bruker, 2005 ▶) *T*_min_ = 0.022, *T*_max_ = 0.356
18721 measured reflections
4094 independent reflections
3477 reflections with *I* > 2σ(*I*)
*R*_int_ = 0.048

#### Refinement

*R*[*F* ^2^ > 2σ(*F* ^2^)] = 0.031
*wR*(*F* ^2^) = 0.078
*S* = 1.01
4094 reflections
172 parameters
H-atom parameters constrained
Δρ_max_ = 0.82 e Å^−3^
Δρ_min_ = −2.14 e Å^−3^

### 

Data collection: *APEX2* (Bruker, 2005 ▶); cell refinement: *SAINT* (Bruker, 2005 ▶); data reduction: *SAINT*; program(s) used to solve structure: *SIR92* (Altomare *et al.*, 1993 ▶); program(s) used to refine structure: *SHELXL97* (Sheldrick, 2008 ▶); molecular graphics: *DIAMOND* (Brandenburg, 2004 ▶); software used to prepare material for publication: *publCIF* (Westrip, 2010 ▶).

## Supplementary Material

Crystal structure: contains datablocks I, global. DOI: 10.1107/S1600536811014085/lr2005sup1.cif

Structure factors: contains datablocks I. DOI: 10.1107/S1600536811014085/lr2005Isup2.hkl

Additional supplementary materials:  crystallographic information; 3D view; checkCIF report

## Figures and Tables

**Table:** 

Pt1—C14	2.045 (5)
Pt1—C13	2.055 (5)
Pt1—C15	2.092 (5)
Pt1—N2	2.160 (4)
Pt1—N1	2.175 (4)
Pt1—I1	2.7755 (5)

**Table:** 

C14—Pt1—C13	85.8 (2)
C13—Pt1—N1	98.62 (18)
C15—Pt1—N1	90.43 (18)
N2—Pt1—N1	76.52 (16)
C15—Pt1—I1	179.94 (17)

**Fig. 1.**
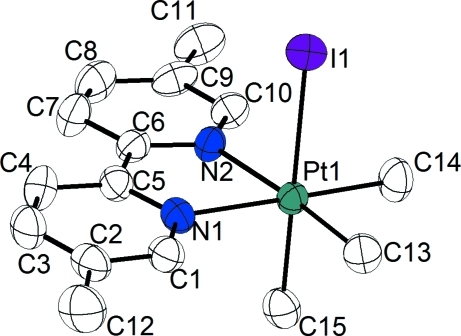
[The asymmetric unit of the title compound with atom labels and 50% probability displacement ellipsoids. Hydrogen atoms are omitted for clarity.]

**Fig. 2.**
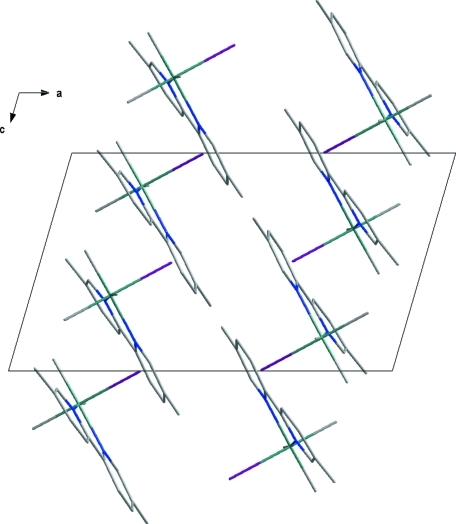
[Packing diagram of the title compound viewed along the b axis. Hydrogen atoms are omitted for clarity.]

## supplementary crystallographic information

### Comment

As a part of a larger project, the title compound and several other compounds based on substituted bipyridines and Pt^IV^ salts were synthesized in an NMR-screening for new Pt^IV^ complexes for potential application in bimetallic MOFs (Metal-Organic Frameworks). Bimetallic MOFs containing bipyridine, Pt(II) (Szeto *et al.*, 2006, 2008) and Pd(II) (Bloch *et al.*, 2010) have previously been reported, but so far no thermally stable bimetallic MOF containing Pt^IV^ is reported in the literature.

The title compound has previously been reported (Clegg *et al.*, 1972) as a part of an extensive NMR-studies to find evidence for *cis* and *trans* influences in trimethylplatinum(IV) compounds. The NMR spectra of the compound is in good accordance with what was reported, and the crystal structure supports the finds with regards to *trans* influences.

### Experimental

The title compound was synthesized by a modified version of the method used by Clegg *et al.* (1972). 5,5'-dimethyl-2,2'-bipyridine (1.2 mg, 0.006 mmol) and Pt^IV^(CH_3_)_3_I  (2.0 mg, 0,005 mmol) was weighed out in an NMR-tube, and 0.5 ml of deuterated benzene (C_6_D_6_) was added. The resulting mixture was heated to 75 °C for 3 days without stirring. A number of NMR-spectra were recorded during the synthesis to monitor the formation of the complex. After the NMR-experiments were finished, the NMR-tube was left at ambient temperature for 7 days, during which crystals of the complex formed.

### Refinement

H-atoms were positioned geometrically at distances of 0.93(CH) and 0.96Å (CH_3_) and refined using a riding model with U_iso_(H)=1.2 U_eq_(C) and U_iso_(H)=1.5 U_eq_(C_methyl_)

### Crystal data

**Table:**

[Pt(CH_3_)_3_I(C_12_H_12_N_2_)]	*F*(000) = 1024
*M**_r_* = 551.32	*D*_x_ = 2.211 Mg m^−^^3^
Monoclinic, *P*2_1_/*c*	Mo *K*α radiation, λ = 0.71073 Å
Hall symbol: -P 2ybc	Cell parameters from 7297 reflections
*a* = 15.354 (3) Å	θ = 2.8–27.6°
*b* = 12.394 (2) Å	µ = 10.33 mm^−^^1^
*c* = 9.0627 (18) Å	*T* = 293 K
β = 106.222 (2)°	Plate, orange
*V* = 1655.8 (6) Å^3^	0.4 × 0.4 × 0.1 mm
*Z* = 4	

### Data collection

**Table:**

Bruker APEXII CCD area-detector diffractometer	4094 independent reflections
Radiation source: sealed tube	3477 reflections with *I* > 2σ(*I*)
graphite	*R*_int_ = 0.048
φ and ω scans	θ_max_ = 29.0°, θ_min_ = 2.2°
Absorption correction: numerical (*SADABS*; Bruker, 2005)	*h* = −19→19
*T*_min_ = 0.022, *T*_max_ = 0.356	*k* = −16→16
18721 measured reflections	*l* = −11→12

### Refinement

**Table:**

Refinement on *F*^2^	Primary atom site location: structure-invariant direct methods
Least-squares matrix: full	Secondary atom site location: difference Fourier map
*R*[*F*^2^ > 2σ(*F*^2^)] = 0.031	Hydrogen site location: inferred from neighbouring sites
*wR*(*F*^2^) = 0.078	H-atom parameters constrained
*S* = 1.01	*w* = 1/[σ^2^(*F*_o_^2^) + (0.045*P*)^2^] where *P* = (*F*_o_^2^ + 2*F*_c_^2^)/3
4094 reflections	(Δ/σ)_max_ = 0.001
172 parameters	Δρ_max_ = 0.82 e Å^−^^3^
0 restraints	Δρ_min_ = −2.14 e Å^−^^3^

### Special details

**Table:**

Experimental. Synthesis of the complex was performed in deuterated solvent.
Geometry. All s.u.'s (except the s.u. in the dihedral angle between two l.s. planes) are estimated using the full covariance matrix. The cell s.u.'s are taken into account individually in the estimation of s.u.'s in distances, angles and torsion angles; correlations between s.u.'s in cell parameters are only used when they are defined by crystal symmetry. An approximate (isotropic) treatment of cell s.u.'s is used for estimating s.u.'s involving l.s. planes.
Refinement. Refinement of *F*^2^ against ALL reflections. The weighted *R*-factor *wR* and goodness of fit *S* are based on *F*^2^, conventional *R*-factors *R* are based on *F*, with *F* set to zero for negative *F*^2^. The threshold expression of *F*^2^ > 2σ(*F*^2^) is used only for calculating *R*-factors(gt) *etc*. and is not relevant to the choice of reflections for refinement. *R*-factors based on *F*^2^ are statistically about twice as large as those based on *F*, and *R*- factors based on ALL data will be even larger.

### Fractional atomic coordinates and isotropic or equivalent isotropic displacement parameters (Å^2^)

**Table:**

	*x*	*y*	*z*	*U*_iso_*/*U*_eq_	
Pt1	0.208561 (12)	0.071224 (14)	0.65323 (2)	0.03239 (8)	
I1	0.34139 (2)	0.03855 (3)	0.50370 (4)	0.04465 (10)	
N2	0.3088 (3)	0.0375 (3)	0.8682 (4)	0.0354 (9)	
C1	0.1397 (3)	−0.1655 (4)	0.5835 (6)	0.0412 (11)	
H1	0.0981	−0.1344	0.4995	0.049*	
C3	0.1999 (4)	−0.3208 (4)	0.7271 (7)	0.0540 (14)	
H3	0.2019	−0.3949	0.7433	0.065*	
C9	0.4219 (4)	0.0869 (5)	1.0999 (6)	0.0459 (13)	
C8	0.4289 (4)	−0.0211 (5)	1.1424 (6)	0.0512 (14)	
H8	0.4691	−0.0412	1.2355	0.061*	
C7	0.3777 (4)	−0.0985 (5)	1.0502 (7)	0.0520 (14)	
H7	0.3831	−0.1707	1.0796	0.062*	
N1	0.1984 (3)	−0.1024 (3)	0.6788 (5)	0.0369 (9)	
C13	0.1095 (4)	0.0898 (4)	0.4489 (6)	0.0440 (12)	
H13A	0.0845	0.0206	0.4125	0.066*	
H13B	0.1353	0.1220	0.3743	0.066*	
H13C	0.0623	0.1356	0.4643	0.066*	
C2	0.1371 (4)	−0.2776 (4)	0.6031 (7)	0.0474 (13)	
C15	0.1083 (4)	0.0958 (4)	0.7657 (6)	0.0451 (12)	
H15A	0.1350	0.0914	0.8748	0.068*	
H15B	0.0623	0.0414	0.7342	0.068*	
H15C	0.0817	0.1658	0.7397	0.068*	
C4	0.2599 (4)	−0.2542 (4)	0.8277 (6)	0.0507 (13)	
H4	0.3021	−0.2835	0.9125	0.061*	
C14	0.2236 (4)	0.2350 (4)	0.6478 (6)	0.0487 (13)	
H14A	0.2700	0.2578	0.7372	0.073*	
H14B	0.1673	0.2694	0.6465	0.073*	
H14C	0.2404	0.2548	0.5571	0.073*	
C11	0.4762 (4)	0.1730 (5)	1.1990 (7)	0.0587 (16)	
H11A	0.5147	0.1412	1.2907	0.088*	
H11B	0.4360	0.2239	1.2259	0.088*	
H11C	0.5127	0.2093	1.1441	0.088*	
C12	0.0671 (5)	−0.3445 (5)	0.4897 (8)	0.0661 (17)	
H12A	0.0305	−0.2984	0.4115	0.099*	
H12B	0.0291	−0.3806	0.5422	0.099*	
H12C	0.0971	−0.3970	0.4433	0.099*	
C5	0.2577 (3)	−0.1439 (4)	0.8034 (6)	0.0381 (10)	
C6	0.3172 (3)	−0.0669 (4)	0.9108 (6)	0.0358 (10)	
C10	0.3605 (3)	0.1121 (4)	0.9595 (5)	0.0395 (10)	
H10	0.3550	0.1837	0.9276	0.047*	

### Atomic displacement parameters (Å^2^)

**Table:**

	*U*^11^	*U*^22^	*U*^33^	*U*^12^	*U*^13^	*U*^23^
Pt1	0.02979 (11)	0.02980 (11)	0.03496 (12)	−0.00090 (6)	0.00471 (8)	0.00234 (6)
I1	0.04027 (19)	0.0491 (2)	0.04544 (19)	−0.00213 (15)	0.01335 (15)	−0.00193 (15)
N2	0.035 (2)	0.036 (2)	0.034 (2)	0.0054 (17)	0.0069 (17)	0.0060 (16)
C1	0.043 (3)	0.035 (3)	0.048 (3)	0.000 (2)	0.018 (2)	−0.001 (2)
C3	0.063 (4)	0.032 (3)	0.072 (4)	0.002 (3)	0.027 (3)	0.006 (3)
C9	0.030 (3)	0.061 (3)	0.042 (3)	0.004 (2)	0.002 (2)	−0.009 (2)
C8	0.046 (3)	0.063 (4)	0.038 (3)	0.009 (3)	0.000 (2)	0.005 (3)
C7	0.051 (3)	0.049 (3)	0.052 (3)	0.014 (3)	0.008 (3)	0.015 (3)
N1	0.040 (2)	0.033 (2)	0.040 (2)	−0.0036 (18)	0.0136 (19)	0.0008 (17)
C13	0.041 (3)	0.044 (3)	0.041 (3)	−0.007 (2)	0.002 (2)	0.000 (2)
C2	0.052 (3)	0.031 (2)	0.068 (4)	−0.005 (2)	0.031 (3)	−0.007 (2)
C15	0.048 (3)	0.037 (3)	0.049 (3)	−0.008 (2)	0.012 (2)	−0.002 (2)
C4	0.059 (3)	0.036 (3)	0.055 (3)	0.010 (3)	0.013 (3)	0.008 (2)
C14	0.053 (3)	0.030 (3)	0.057 (3)	−0.003 (2)	0.006 (3)	0.002 (2)
C11	0.044 (3)	0.075 (4)	0.048 (3)	0.002 (3)	−0.001 (3)	−0.014 (3)
C12	0.069 (4)	0.046 (3)	0.084 (5)	−0.012 (3)	0.023 (4)	−0.012 (3)
C5	0.043 (3)	0.034 (2)	0.041 (2)	0.004 (2)	0.018 (2)	0.003 (2)
C6	0.032 (2)	0.043 (3)	0.033 (2)	0.0062 (19)	0.009 (2)	0.0047 (19)
C10	0.036 (3)	0.042 (3)	0.037 (2)	0.000 (2)	0.006 (2)	−0.003 (2)

### Geometric parameters (Å, °)

**Table:**

Pt1—C14	2.045 (5)	N1—C5	1.339 (6)
Pt1—C13	2.055 (5)	C13—H13A	0.9600
Pt1—C15	2.092 (5)	C13—H13B	0.9600
Pt1—N2	2.160 (4)	C13—H13C	0.9600
Pt1—N1	2.175 (4)	C2—C12	1.511 (8)
Pt1—I1	2.7755 (5)	C15—H15A	0.9600
N2—C10	1.342 (6)	C15—H15B	0.9600
N2—C6	1.346 (6)	C15—H15C	0.9600
C1—N1	1.317 (6)	C4—C5	1.384 (7)
C1—C2	1.402 (7)	C4—H4	0.9300
C1—H1	0.9300	C14—H14A	0.9600
C3—C2	1.369 (8)	C14—H14B	0.9600
C3—C4	1.375 (8)	C14—H14C	0.9600
C3—H3	0.9300	C11—H11A	0.9600
C9—C8	1.388 (8)	C11—H11B	0.9600
C9—C10	1.392 (7)	C11—H11C	0.9600
C9—C11	1.490 (8)	C12—H12A	0.9600
C8—C7	1.367 (9)	C12—H12B	0.9600
C8—H8	0.9300	C12—H12C	0.9600
C7—C6	1.400 (7)	C5—C6	1.482 (7)
C7—H7	0.9300	C10—H10	0.9300
			
C14—Pt1—C13	85.8 (2)	H13B—C13—H13C	109.5
C14—Pt1—C15	88.3 (2)	C3—C2—C1	116.9 (5)
C13—Pt1—C15	87.9 (2)	C3—C2—C12	123.2 (5)
C14—Pt1—N2	99.02 (19)	C1—C2—C12	119.8 (5)
C13—Pt1—N2	175.08 (17)	Pt1—C15—H15A	109.5
C15—Pt1—N2	91.41 (19)	Pt1—C15—H15B	109.5
C14—Pt1—N1	175.34 (19)	H15A—C15—H15B	109.5
C13—Pt1—N1	98.62 (18)	Pt1—C15—H15C	109.5
C15—Pt1—N1	90.43 (18)	H15A—C15—H15C	109.5
N2—Pt1—N1	76.52 (16)	H15B—C15—H15C	109.5
C14—Pt1—I1	91.72 (16)	C3—C4—C5	120.3 (5)
C13—Pt1—I1	92.07 (16)	C3—C4—H4	119.8
C15—Pt1—I1	179.94 (17)	C5—C4—H4	119.8
N2—Pt1—I1	88.65 (10)	Pt1—C14—H14A	109.5
N1—Pt1—I1	89.55 (11)	Pt1—C14—H14B	109.5
C10—N2—C6	119.5 (4)	H14A—C14—H14B	109.5
C10—N2—Pt1	124.9 (3)	Pt1—C14—H14C	109.5
C6—N2—Pt1	115.6 (3)	H14A—C14—H14C	109.5
N1—C1—C2	122.9 (5)	H14B—C14—H14C	109.5
N1—C1—H1	118.5	C9—C11—H11A	109.5
C2—C1—H1	118.5	C9—C11—H11B	109.5
C2—C3—C4	119.8 (5)	H11A—C11—H11B	109.5
C2—C3—H3	120.1	C9—C11—H11C	109.5
C4—C3—H3	120.1	H11A—C11—H11C	109.5
C8—C9—C10	116.7 (5)	H11B—C11—H11C	109.5
C8—C9—C11	122.5 (5)	C2—C12—H12A	109.5
C10—C9—C11	120.8 (5)	C2—C12—H12B	109.5
C7—C8—C9	121.4 (5)	H12A—C12—H12B	109.5
C7—C8—H8	119.3	C2—C12—H12C	109.5
C9—C8—H8	119.3	H12A—C12—H12C	109.5
C8—C7—C6	118.6 (5)	H12B—C12—H12C	109.5
C8—C7—H7	120.7	N1—C5—C4	119.6 (5)
C6—C7—H7	120.7	N1—C5—C6	117.1 (4)
C1—N1—C5	120.3 (4)	C4—C5—C6	123.3 (5)
C1—N1—Pt1	125.0 (3)	N2—C6—C7	121.0 (5)
C5—N1—Pt1	114.7 (3)	N2—C6—C5	116.0 (4)
Pt1—C13—H13A	109.5	C7—C6—C5	123.0 (5)
Pt1—C13—H13B	109.5	N2—C10—C9	122.8 (5)
H13A—C13—H13B	109.5	N2—C10—H10	118.6
Pt1—C13—H13C	109.5	C9—C10—H10	118.6
H13A—C13—H13C	109.5		
			
C14—Pt1—N2—C10	0.3 (4)	N1—C1—C2—C3	−0.7 (8)
C15—Pt1—N2—C10	−88.2 (4)	N1—C1—C2—C12	179.4 (5)
N1—Pt1—N2—C10	−178.3 (4)	C2—C3—C4—C5	−0.7 (8)
I1—Pt1—N2—C10	91.9 (4)	C1—N1—C5—C4	3.0 (7)
C14—Pt1—N2—C6	179.4 (3)	Pt1—N1—C5—C4	−177.3 (4)
C15—Pt1—N2—C6	90.9 (3)	C1—N1—C5—C6	−175.8 (4)
N1—Pt1—N2—C6	0.8 (3)	Pt1—N1—C5—C6	3.9 (5)
I1—Pt1—N2—C6	−89.1 (3)	C3—C4—C5—N1	−1.8 (8)
C10—C9—C8—C7	0.1 (8)	C3—C4—C5—C6	177.0 (5)
C11—C9—C8—C7	179.4 (6)	C10—N2—C6—C7	1.3 (7)
C9—C8—C7—C6	−0.2 (9)	Pt1—N2—C6—C7	−177.9 (4)
C2—C1—N1—C5	−1.8 (8)	C10—N2—C6—C5	−180.0 (4)
C2—C1—N1—Pt1	178.6 (4)	Pt1—N2—C6—C5	0.9 (5)
C13—Pt1—N1—C1	−2.1 (4)	C8—C7—C6—N2	−0.4 (8)
C15—Pt1—N1—C1	85.8 (4)	C8—C7—C6—C5	−179.1 (5)
N2—Pt1—N1—C1	177.1 (4)	N1—C5—C6—N2	−3.2 (6)
I1—Pt1—N1—C1	−94.1 (4)	C4—C5—C6—N2	178.0 (5)
C13—Pt1—N1—C5	178.2 (4)	N1—C5—C6—C7	175.5 (5)
C15—Pt1—N1—C5	−93.9 (4)	C4—C5—C6—C7	−3.3 (8)
N2—Pt1—N1—C5	−2.5 (3)	C6—N2—C10—C9	−1.4 (7)
I1—Pt1—N1—C5	86.2 (3)	Pt1—N2—C10—C9	177.6 (4)
C4—C3—C2—C1	1.9 (8)	C8—C9—C10—N2	0.8 (8)
C4—C3—C2—C12	−178.2 (5)	C11—C9—C10—N2	−178.5 (5)
